# Supplementary figures and images for: Nuclear NAD+-biosynthetic enzyme NMNAT1 facilitates development and early survival of retinal neurons
Source: eLife. 2021 Dec 8;10:e71185. doi: 10.7554/eLife.71185 (PMC8754432; doi:10.7554/eLife.71185)

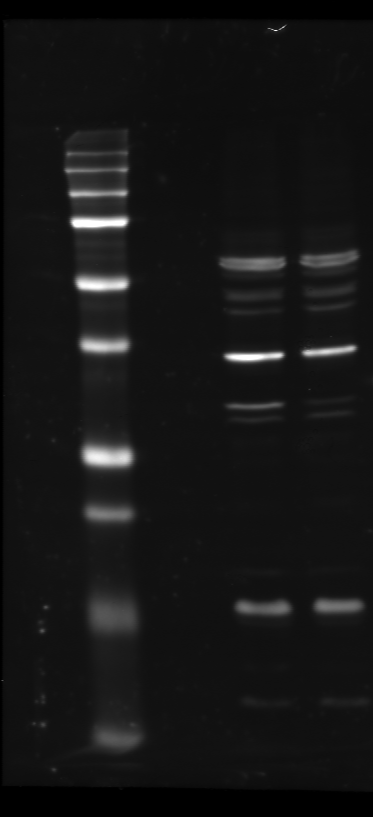

Supplement: Figure 1—figure supplement 1—source data 1. [file elife-71185-fig1-figsupp1-data1.zip › Figure1S1_SourceData1.tif]

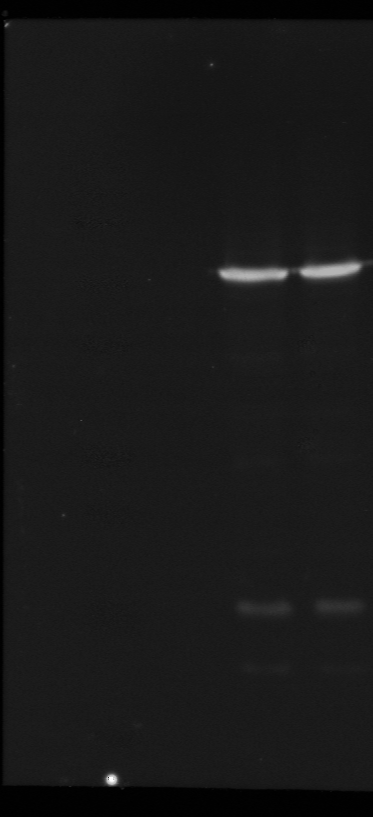

Supplement: Figure 1—figure supplement 1—source data 2. [file elife-71185-fig1-figsupp1-data2.zip › Figure1S1_SourceData2.tif]

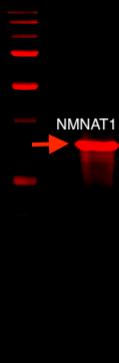

Supplement: Figure 1—figure supplement 1—source data 3. — Band corresponding to NMNAT1 is clearly labeled. [file elife-71185-fig1-figsupp1-data3.zip › Figure1S1_SourceData3.tif]

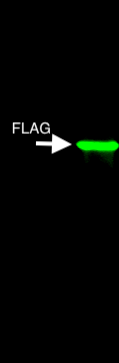

Supplement: Figure 1—figure supplement 1—source data 4. — Band corresponding to FLAG is clearly labeled. [file elife-71185-fig1-figsupp1-data4.zip › Figure1S1_SourceData4.tif]

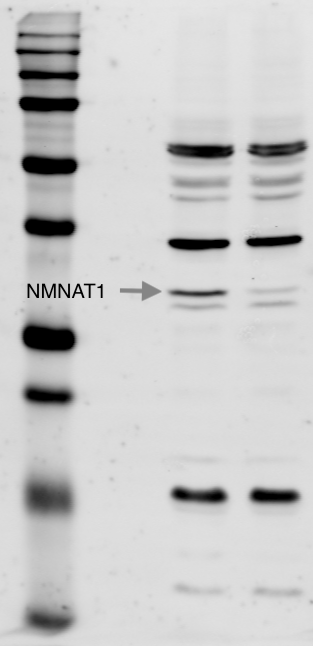

Supplement: Figure 1—figure supplement 1—source data 5. — Band corresponding to NMNAT1 is clearly labeled. [file elife-71185-fig1-figsupp1-data5.zip › Figure1S1_SourceData5.tif]

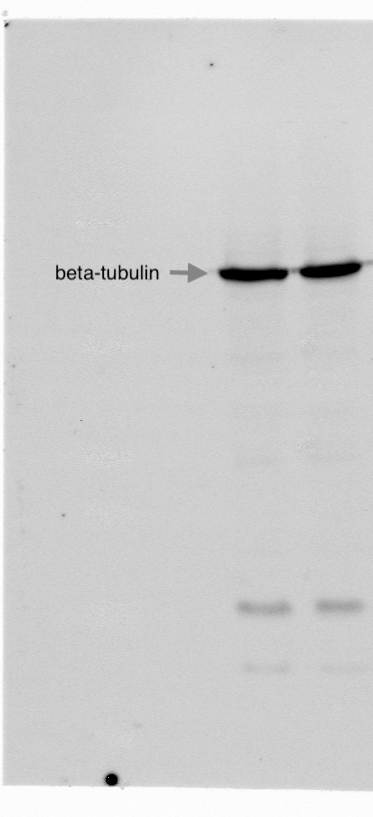

Supplement: Figure 1—figure supplement 1—source data 6. — Band corresponding to beta-tubulin is clearly labeled. [file elife-71185-fig1-figsupp1-data6.zip › Figure1S1_SourceData6.tif]

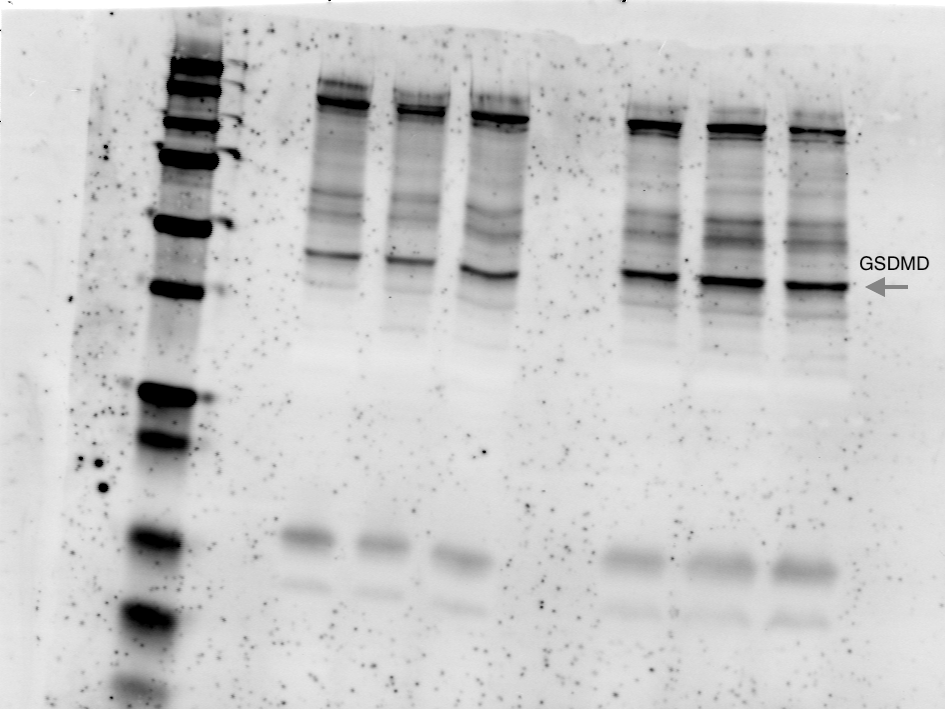

Supplement: Figure 4—figure supplement 1—source data 2. — Arrow denotes bands corresponding to GSDMD. [file elife-71185-fig4-figsupp1-data2.zip › Figure4S1_SourceData2.tif]

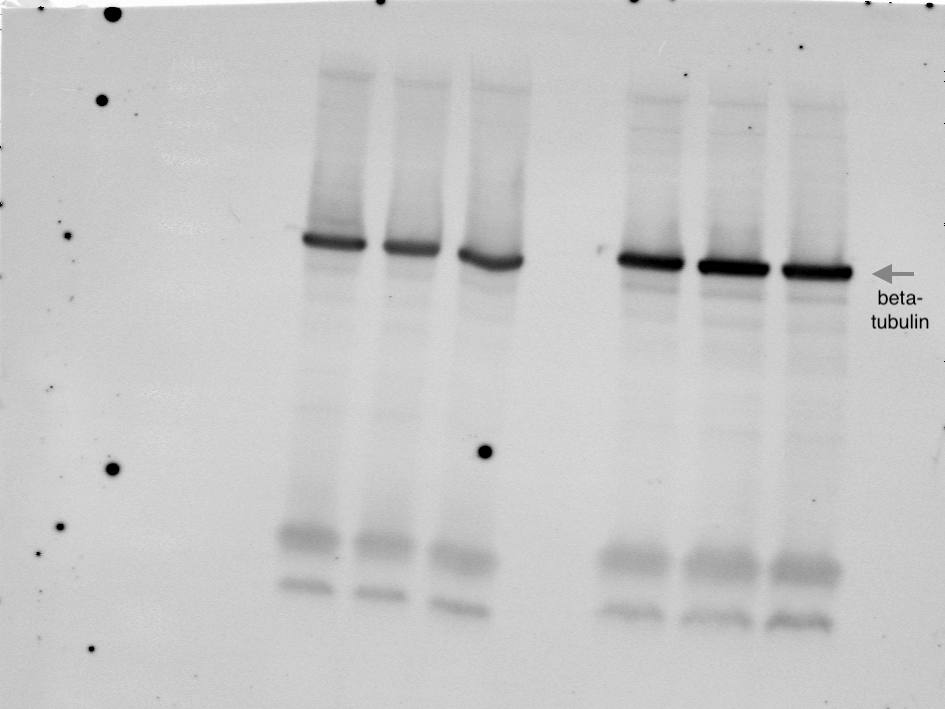

Supplement: Figure 4—figure supplement 1—source data 3. — Arrow denotes bands corresponding to beta-tubulin. [file elife-71185-fig4-figsupp1-data3.zip › Figure4S1_SourceData3.tif]

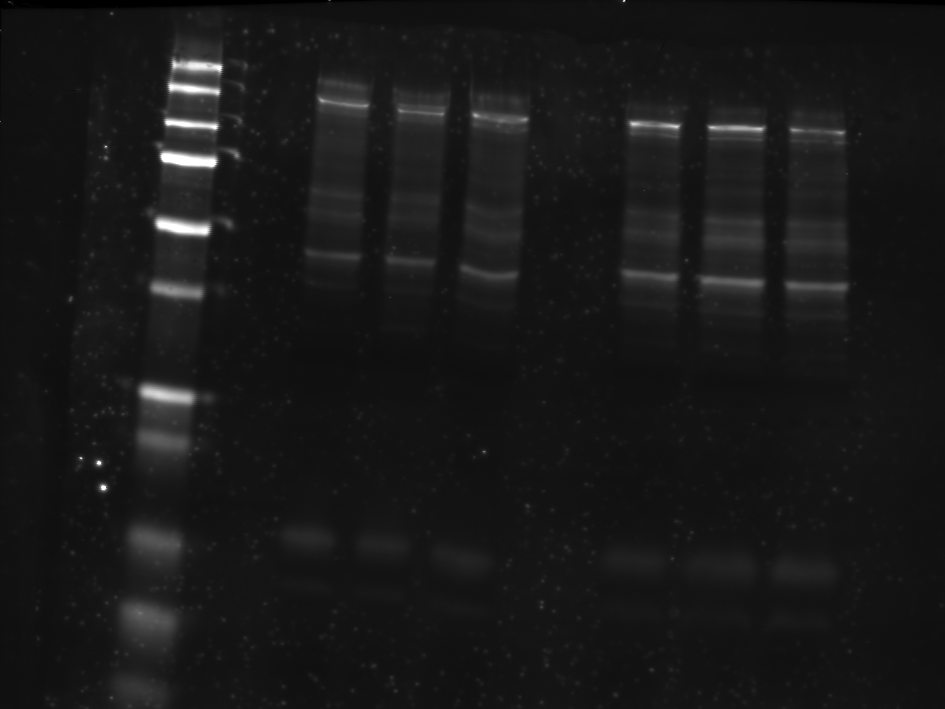

Supplement: Figure 4—figure supplement 1—source data 4. [file elife-71185-fig4-figsupp1-data4.zip › Figure4S1_SourceData4.tif]

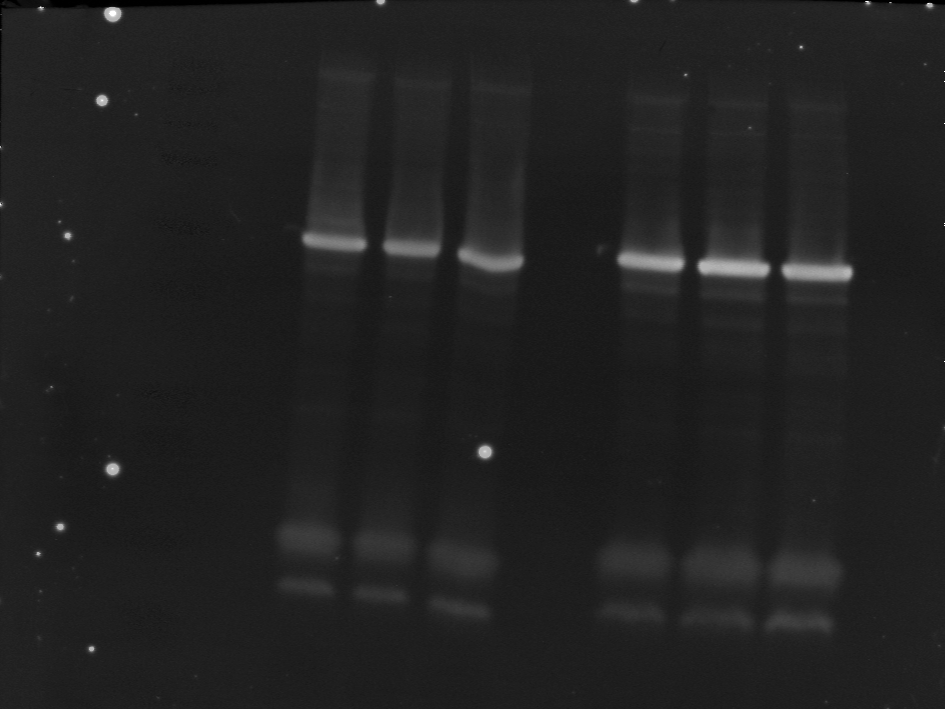

Supplement: Figure 4—figure supplement 1—source data 5. [file elife-71185-fig4-figsupp1-data5.zip › Figure4S1_SourceData5.tif]
