# Supplementary material for: Nuclear NAD+-biosynthetic enzyme NMNAT1 facilitates development and early survival of retinal neurons
Source: eLife. 2021 Dec 8;10:e71185. doi: 10.7554/eLife.71185 (PMC8754432; doi:10.7554/eLife.71185)
Supplement: Supplementary file 1. [file elife-71185-supp1.docx]

| **Feature** | **Forward Primer Sequence** | **Reverse Primer Sequence** |
| --- | --- | --- |
| *Six3*-Cre | 5’-CCTGGAAAATGCTTCTGTCCG-3’ | 5’-CAGGGTGTTATAAGCAATCCC-3’ |
| *Nmnat1* 5’ loxP | 5’-TCGGAGTGTATCCTTGGAGT-3’ | 5’-ACCAAGCTTTCAGCACATGG-3’ |
| *Nmnat1* 3’ loxP | 5’-CCCAGTCACTAAGACATTCAA-3’ | 5’-GACCCTCCTAGGCAAATATA-3’ |
| *Nmnat1* (RT-qPCR) | 5’-CTTTTAACCCCATCACCAACATGC-3’ | 5’-CCTTTCTTCTTGTACGCATCACC-3’ |
| *Nmnat2* | 5’-CTTTTGTAGATGAGAACGCCAACC-3’ | 5’-CCAACAATCACTTCCATATCTGCC-3’ |
| *Nmnat3* | 5’-AAGACACCATCAGCCTCTGC-3’ | 5’-CCAAGCCGAACTTCTCCACT-3’ |
| *Cep290* | 5’-AAGGTACTGAGAAAATTGTTGCCG-3’ | 5’-TGTAGTCTCTTCCCAGTTTCTTCG-3’ |
| *Prom1* | 5’-TGAGACCCAAGATACCTTCAATGC-3’ | 5’-AGACTATGATTTCTGGCTCCTTGG-3’ |
| *Gnat1* | 5’-GGAGAAGAAGCTGAAAGAGGATGC-3’ | 5’-AGAGTGTTGCCGTAGATGATGG-3’ |
| *Rp1* | 5’-CAAGTTACCAGGAATCTCTCATCG-3’ | 5’-TCTAAGGCCAAGTAATTCTCAGGG-3’ |
| *Hmbs* | 5’-GTTTACCAAGGAGCTAGAAAACGC-3’ | 5’-GTGAAAGACAACAGCATCACAAGG-3’ |
| *Ppia* | 5’-GGATTTGGCTATAAGGGTTCCTCC-3’ | 5’-GTTCTCATCCTCAAATTTCTCTCCG-3’ |
| *Ywhaz* | 5’- GTTGTAGGAGCCCGTAGGTCATCG-3’ | 5’- GCTTTCTGGTTGCGAAGCATTGGG-3’ |

**Supplementary Tables- Sokolov et al.**

**Supplementary Table 1. Primer sequences for genotyping and RT-qPCR experiments.**

| Metabolite | HMDB | Positive/ Negative mode | Q1 Mass (Da) | Q3 Mass (Da) | Declustering Potential (Volts) | Collision Energy (Volts) | Retention time (RT) |
| --- | --- | --- | --- | --- | --- | --- | --- |
| 1-Methyladenosine | HMDB0003331 | Pos | 282.1 | 150.1 | 48 | 35 | 1.85 |
| 2-Methylbutyroylcarnitine | HMDB0000378 | Pos | 246.2 | 85.1 | 100 | 43 | 1.3 |
| D-2-Hydroxyglutaric acid | HMDB0000606 | Pos | 149.1 | 77 | 100 | 43 | 1.37 |
| 3-Aminoisobutanoic acid | HMDB0003911 | Pos | 104.1 | 58 | 55 | 38 | 1.37 |
| 3-Hydroxybutyric acid | HMDB0000357 | Pos | 105.1 | 45 | 20 | 47 | 1.3 |
| Hydroxykynurenine | HMDB0000732 | Neg | 223 | 75 | -150 | -55 | 0.3 |
| p-Aminobenzoic acid | HMDB0001392 | Pos | 138.1 | 120 | 63 | 24 | 0.46 |
| 4-Hydroxyphenylpyruvic acid | HMDB0000707 | Neg | 179.1 | 97.1 | -40 | -17 | 4.08 |
| 4-Hydroxyproline | HMDB0000725 | Pos | 132.1 | 86 | 60 | 17 | 2.63 |
| N-Acetyl-L-aspartic acid | HMDB0000812 | Pos | 176.1 | 74 | 50 | 20 | 3.4 |
| Acetyl-CoA | HMDB0001206 | Pos | 810.1 | 303.1 | 116 | 46 | 3.98 |
| N-Alpha-acetyllysine | HMDB0000446 | Pos | 189.1 | 84.1 | 50 | 32 | 5 |
| N-acetyltryptophan | HMDB0013713 | Pos | 247.2 | 146 | 75 | 35 | 0.42 |
| N-Acetylasparagine | HMDB0006028 | Pos | 175.1 | 70.1 | 55 | 24 | 3.1 |
| N-Acetylglutamic acid | HMDB0001138 | Pos | 190.1 | 84.1 | 51 | 20 | 3.02 |
| cis-Aconitic acid | HMDB0000072 | Neg | 173 | 85 | -37 | -18 | 3.66 |
| Adenine | HMDB0000034 | Neg | 134 | 107 | -92 | -25 | 0.73 |
| Adenosine | HMDB0000050 | Pos | 268.1 | 136 | 95 | 38 | 0.79 |
| Adipic acid | HMDB0000448 | Neg | 145 | 83 | -50 | -17 | 3.14 |
| ADP | HMDB0001341 | Neg | 426 | 79.1 | -65 | -84 | 4.6 |
| Oxoglutaric acid | HMDB0000208 | Neg | 145 | 101 | -56 | -11 | 3.02 |
| L-Alanine | HMDB0000161 | Pos | 90 | 44 | 50 | 20 | 2.35 |
| Aminoadipic acid | HMDB0000510 | neg | 160.1 | 116.1 | -50 | -20 | 3.75 |
| Gamma-Aminobutyric acid | HMDB0000112 | Pos | 104.1 | 87 | 57 | 13 | 2.57 |
| Adenosine monophosphate | HMDB0000045 | Neg | 346 | 134 | -82 | -46 | 3.76 |
| L-Arginine | HMDB0000517 | Pos | 175.1 | 70.1 | 45 | 33 | 4.28 |
| Argininosuccinic acid | HMDB0000052 | Pos | 291.1 | 69.9 | 130 | 65 | 4.6 |
| Ascorbic acid | HMDB0000044 | Neg | 175 | 87 | -63 | -30 | 2.3 |
| L-Asparagine | HMDB0000168 | Pos | 133.1 | 70.1 | 55 | 24 | 2.94 |
| L-Aspartic acid | HMDB0000191 | Pos | 134.1 | 74 | 50 | 20 | 3.49 |
| Adenosine triphosphate | HMDB0000538 | Pos | 507.9 | 136.1 | 14 | 60 | 5.69 |
| Azelaic acid | HMDB0000784 | Neg | 187 | 97.1 | -73 | -25 | 2.48 |
| Betaine | HMDB0000043 | Pos | 118.1 | 58 | 166 | 56 | 1.67 |
| Biotin | HMDB0000030 | Neg | 243.1 | 200 | -84 | -23 | 1.62 |
| Butyrylcarnitine | HMDB0002013 | Pos | 232.2 | 85.1 | 72 | 30 | 1.5 |
| Cadaverine | HMDB0002322 | Pos | 103 | 77 | 190 | 25 | 1.02 |
| Cyclic AMP | HMDB0000058 | Neg | 328 | 134 | -87 | -32 | 2.28 |
| Carbamoyl phosphate | HMDB0001096 | Neg | 151 | 108 | -105 | -25 | 1.04 |
| L-Carnitine | HMDB0000062 | Pos | 163.1 | 85 | 100 | 15 | 2.6 |
| Carnosine | HMDB0000033 | Pos | 227.1 | 110.1 | 157 | 32 | 3.61 |
| Cyclic GMP | HMDB0001314 | Neg | 344 | 150 | -37 | -30 | 2.75 |
| Choline | HMDB0000097 | Pos | 104.1 | 60.1 | 95 | 37 | 1.51 |
| Citraconic acid | HMDB0000634 | Neg | 129 | 85 | -21 | -12 | 3.63 |
| Citric acid | HMDB0000094 | Neg | 191.1 | 87 | -73 | -25 | 2.39 |
| Citrulline | HMDB0000904 | Neg | 174.1 | 131.1 | -32 | -20 | 3.28 |
| Coenzyme A | HMDB0001423 | Pos | 768.1 | 261 | 34 | 47 | 4.3 |
| Creatine | HMDB0000064 | Pos | 132.1 | 90 | 170 | 17 | 2.66 |
| Creatinine | HMDB0000562 | Pos | 114 | 44 | 71 | 33 | 0.8 |
| L-Cystine | HMDB0000192 | Neg | 239 | 74 | -50 | -25 | 4.26 |
| Cytidine | HMDB0000089 | Pos | 244 | 112.1 | 50 | 30 | 1.39 |
| Cytosine | HMDB0000630 | Pos | 112 | 95 | 97 | 23 | 0.87 |
| Decanoylcarnitine | HMDB0000651 | Pos | 316.3 | 85.1 | 70 | 25 | 0.6 |
| Dihydroxyacetone phosphate | HMDB0001473 | Neg | 169 | 79 | -38 | 37 | 4.11 |
| Sphinganine | HMDB0000269 | Pos | 302.3 | 81 | 160 | 47 | 1.1 |
| Erythritol | HMDB0002994 | Pos | 123 | 80 | 40 | 33 | 0.38 |
| D-Erythrose 4-phosphate | HMDB0001321 | Neg | 199 | 97 | -160 | -15 | 4.1 |
| FAD | HMDB0001248 | Neg | 784.1 | 437.1 | 52.31 | 43 | 3.8 |
| Fructose 1,6-bisphosphate | HMDB0001058 | Neg | 339.1 | 79 | -56 | -75 | 8.74 |
| Glucose 1-phosphate | HMDB0001586 | Pos | 259 | 79 | -85 | -60 | 4.17 |
| Glucose 6-phosphate | HMDB0001401 | Neg | 259 | 79 | -46 | -62 | 4.11 |
| Guanosine diphosphate | HMDB0001201 | Pos | 444 | 152 | 65 | 26 | 5 |
| Geranyl-PP | HMDB0001285 | Neg | 313.1 | 185.1 | -58 | -45 | 0.27 |
| D-Glucose | HMDB0000122 | Neg | 179 | 89 | -50 | -15 | 2.07 |
| L-Glutamic acid | HMDB0000148 | Pos | 148.1 | 84.1 | 51 | 20 | 3.3 |
| L-Glutamine | HMDB0000641 | Pos | 147.1 | 84.1 | 45 | 23 | 3.03 |
| Glutaric acid | HMDB0000661 | Neg | 131 | 87 | -45 | -16 | 3.41 |
| Glycine | HMDB0000123 | Pos | 76 | 30.1 | 30 | 16 | 2.57 |
| Guanosine monophosphate | HMDB0001397 | Neg | 362.1 | 79 | -41 | -61 | 4.22 |
| Glutathione | HMDB0000125 | Neg | 306 | 143.1 | -61 | -26 | 3.8 |
| Oxidized glutathione | HMDB0003337 | Pos | 613 | [231](https://www.metaboanalyst.ca/MetaboAnalyst/Secure/process/NameMapView.xhtml) | 16 | 42 | 5.56 |
| Guanosine triphosphate | HMDB0001273 | Neg | 521.9 | 159 | -75 | -57 | 5.2 |
| Guanine | HMDB0000132 | Pos | 152 | 110 | 20 | 28 | 1.21 |
| Guanosine | HMDB0000133 | Neg | 282.1 | 150 | -67 | -33 | 1.64 |
| Heptadecanoic acid | HMDB0002259 | Neg | 269.1 | 135.1 | -77 | -35 | 1.05 |
| Hexanoylcarnitine | HMDB0000705 | Pos | 260.2 | 85.1 | 80 | 25 | 0.9 |
| Histamine | HMDB0000870 | Pos | 112 | 95 | 65 | 18 | 2.41 |
| L-Histidine | HMDB0000177 | Pos | 156.1 | 110 | 50 | 22 | 3.35 |
| L-Homoserine | HMDB0000719 | Pos | 120.1 | 74 | 37 | 16 | 3.04 |
| Hypotaurine | HMDB0000965 | Neg | 108.1 | 64 | -40 | -17 | 2.44 |
| Hypoxanthine | HMDB0000157 | Neg | 135 | 65 | -108 | -37 | 0.8 |
| Inosinic acid | HMDB0000175 | Neg | 347 | 79 | -118 | -86 | 4 |
| Inosine | HMDB0000195 | Neg | 267 | 135 | -123 | -30 | 1.1 |
| Isopentenyl pyrophosphate | HMDB0001347 | Neg | 245.1 | 79 | -60 | -50 | 2.7 |
| Isobutyryl-L-carnitine | HMDB0000736 | Pos | 232.1 | 85.1 | 70 | 30 | 1.5 |
| L-Kynurenine | HMDB0000684 | Neg | 207.1 | 144 | -58 | -33 | 1.59 |
| L-Lactic acid | HMDB0000190 | Neg | 89 | 43 | -60 | -17 | 1.13 |
| L-Leucine | HMDB0000687 | Pos | 132.1 | 86 | 60 | 16 | 1.65 |
| L-Lysine | HMDB0000182 | Pos | 147.1 | 84 | 38 | 22 | 4.23 |
| L-Malic acid | HMDB0000156 | Neg | 133 | 115 | -50 | -14 | 3.55 |
| Maleic acid | HMDB0000176 | Neg | 115 | 71 | -34 | -13 | 0.54 |
| Malonyl-CoA | HMDB0001175 | Pos | 854.1 | 347.1 | 75 | 45 | 4.97 |
| L-Methionine | HMDB0000696 | Pos | 150.1 | 61 | 27 | 43 | 1.65 |
| Methylmalonic acid | HMDB0000202 | Pos | 119 | 65 | 70 | 37 | 1.23 |
| myo-Inositol | HMDB0000211 | Neg | 179 | 87 | -105 | -24 | 2.9 |
| Tetradecanoylcarnitine | HMDB0005066 | Pos | 372.4 | 85.1 | 70 | 25 | 0.4 |
| Acetylglycine | HMDB0000532 | Neg | 116 | 74 | -37 | -14 | 1.92 |
| 1-Methylnicotinamide | HMDB0000699 | Pos | 137 | 78 | 110 | 37 | 1.63 |
| NAD | HMDB0000902 | Pos | 664 | 136 | 27 | 48 | 4.33 |
| NADH | HMDB0001487 | Neg | 664 | 397 | -7 | -46 | 3.91 |
| NADP | HMDB0000217 | Pos | 744 | 136 | 9 | 78 | 5.36 |
| NADPH | HMDB0000221 | Neg | 744 | 408 | -35 | -52 | 5.1 |
| Niacinamide | HMDB0001406 | Pos | 123 | 80 | 136 | 20 | 0.41 |
| Nicotinic acid | HMDB0001488 | Neg | 122 | 78 | -50 | -15 | 1.31 |
| Nicotinamide ribotide | HMDB0000229 | Pos | 335 | 123.1 | 40 | 25 | 4.7 |
| Nicotinamide riboside | HMDB00855 | Pos | 256.1 | 123 | 68 | 18 | 2.25 |
| L-Acetylcarnitine | HMDB0000201 | Pos | 204.1 | 85 | 71 | 30 | 1.86 |
| L-Octanoylcarnitine | HMDB0000791 | Pos | 288.3 | 85.1 | 100 | 25 | 0.7 |
| Ophthalmic acid | HMDB0005765 | Pos | 290.1 | 58 | 139 | 56 | 3.66 |
| Ornithine | HMDB0000214 | Pos | 133 | 70 | 47 | 23 | 4.3 |
| Oxalic acid | HMDB0002329 | Neg | 89 | 61 | -45 | -10 | 5.06 |
| Oxalacetic acid | HMDB0000223 | Neg | 131 | 87 | -30 | -11 | 3.79 |
| L-Palmitoylcarnitine | HMDB0000222 | Pos | 400.4 | 85.1 | 30 | 40 | 0.4 |
| Palmityl-CoA | HMDB0001338 | Pos | 1006.3 | 499.5 | 45 | 53 | 3.26 |
| Pantothenic acid | HMDB0000210 | Neg | 218.1 | 71 | -70 | -41 | 1.13 |
| L-Phenylalanine | HMDB0000159 | Pos | 166.1 | 120 | 50 | 18 | 1.37 |
| Phosphocreatine | HMDB0001511 | Neg | 210 | 79 | -42 | -49 | 4.42 |
| Phosphoenolpyruvic acid | HMDB0000263 | Neg | 167.1 | 79 | -38 | -20 | 3.95 |
| L-Proline | HMDB0000162 | Pos | 116.1 | 70.1 | 70,120 | 44 | 2.1 |
| Propionylcarnitine | HMDB0000824 | Pos | 218.1 | 85.1 | 60 | 24 | 1.9 |
| Pyroglutamic acid | HMDB0000267 | Pos | 130.1 | 84 | 100 | 20 | 2.02 |
| Quinic acid | HMDB0003072 | Neg | 191.1 | 85 | -60 | -33 | 2.54 |
| Riboflavin | HMDB0000244 | Pos | 377.1 | 243.1 | 14 | 29 | 1.07 |
| D-Ribulose 5-phosphate | HMDB0000618 | Neg | 229 | 79 | -70 | -45 | 3.43 |
| S-Adenosylmethionine | HMDB0001185 | Pos | 192.1 | 61 | 27 | 43 | 0.48 |
| L-Serine | HMDB0000187 | Pos | 106 | 60 | 40 | 22 | 2.83 |
| Spermine | HMDB0001256 | Pos | 203.1 | 129 | 33 | 26 | 1.9 |
| Stearoylcarnitine | HMDB0000848 | Pos | 428.5 | 85.1 | 70 | 25 | 0.3 |
| Succinic acid | HMDB0000254 | Neg | 117 | 73 | -54 | -16 | 2.92 |
| Taurine | HMDB0000251 | Pos | 126 | 108 | 200 | 15 | 1.8 |
| Thiamine | HMDB0000235 | Pos | 265 | 122.1 | 67 | 40 | 2.1 |
| L-Threonine | HMDB0000167 | Pos | 120.1 | 102 | 50 | 10 | 2.5 |
| Trimethylamine N-oxide | HMDB0000925 | Pos | 76 | 58 | 117 | 43 | 2.3 |
| Trigonelline | HMDB0000875 | Pos | 138 | 92 | 60 | 27 | 1.81 |
| L-Tryptophan | HMDB0000929 | Pos | 205 | 146 | 75 | 35 | 1.79 |
| L-Tyrosine | HMDB0000158 | Pos | 182.1 | 136 | 40 | 17 | 1.92 |
| Uridine diphosphate glucose | HMDB0000286 | Pos | 611 | 499 | 40 | 29 | 4.59 |
| Uracil | HMDB0000300 | Neg | 111 | 42 |  | -37 | 0.53 |
| Uric acid | HMDB0000289 | Neg | 167 | 124 | -86 | -19 | 2.6 |
| Uridine | HMDB0000296 | Pos | 245 | 113 | 23 | 51 | 1.4 |
| L-Valine | HMDB0000883 | Pos | 118.1 | 72 | 60 | 14 | 1.76 |
| Xanthine | HMDB0000292 | Neg | 151 | 108 | -70 | -23 | 1.1 |
| Xanthosine | HMDB0000299 | Neg | 283.1 | 151 | -92 | -28 | 1.66 |
| Xanthurenic acid | HMDB0000881 | Neg | 204 | 160 | -67 | -19 | 1.15 |

**Supplementary Table 2. Mass spectrometry standards and parameters.**
